# Strain-specific morphological response of the dominant calcifying phytoplankton species *Emiliania huxleyi* to salinity change

**DOI:** 10.1371/journal.pone.0246745

**Published:** 2021-02-11

**Authors:** Christina Gebühr, Rosie M. Sheward, Jens O. Herrle, Jörg Bollmann

**Affiliations:** 1 Institute of Geosciences, Goethe-University Frankfurt, Frankfurt am Main, Germany; 2 Biodiversity and Climate Research Centre (BIK-F), Frankfurt am Main, Germany; 3 Department of Earth Sciences, University of Toronto, Toronto, Ontario, Canada; Victoria University of Wellington, NEW ZEALAND

## Abstract

The future physiology of marine phytoplankton will be impacted by a range of changes in global ocean conditions, including salinity regimes that vary spatially and on a range of short- to geological timescales. Coccolithophores have global ecological and biogeochemical significance as the most important calcifying marine phytoplankton group. Previous research has shown that the morphology of their exoskeletal calcified plates (coccoliths) responds to changing salinity in the most abundant coccolithophore species, *Emiliania huxleyi*. However, the extent to which these responses may be strain-specific is not well established. Here we investigated the growth response of six strains of *E*. *huxleyi* under low (ca. 25) and high (ca. 45) salinity batch culture conditions and found substantial variability in the magnitude and direction of response to salinity change across strains. Growth rates declined under low and high salinity conditions in four of the six strains but increased under both low and high salinity in strain RCC1232 and were higher under low salinity and lower under high salinity in strain PLYB11. When detailed changes in coccolith and coccosphere size were quantified in two of these strains that were isolated from contrasting salinity regimes (coastal Norwegian low salinity of ca. 30 and Mediterranean high salinity of ca. 37), the Norwegian strain showed an average 26% larger mean coccolith size at high salinities compared to low salinities. In contrast, coccolith size in the Mediterranean strain showed a smaller size trend (11% increase) but severely impeded coccolith formation in the low salinity treatment. Coccosphere size similarly increased with salinity in the Norwegian strain but this trend was not observed in the Mediterranean strain. Coccolith size changes with salinity compiled for other strains also show variability, strongly suggesting that the effect of salinity change on coccolithophore morphology is likely to be strain specific. We propose that physiological adaptation to local conditions, in particular strategies for plasticity under stress, has an important role in determining ecotype responses to salinity.

## Introduction

Changes in global climate are altering many of the fundamental physical and chemical properties of the oceans that regulate the productivity and ecology of marine phytoplankton groups. The response of the single-celled marine phytoplankton group coccolithophores to changes in surface ocean conditions is of particular interest: as major pelagic producers of both biomass and inorganic carbon (calcium carbonate) [1,2] they are key components of marine primary production, ocean biogeochemical cycles, and biogenic-climate feedbacks [3]. The export of their mineralized exoskeletons from the photic zone to ocean sediments forms a remarkably complete fossil record [4] that archives the integrated physiological and ecological effects of changes in marine conditions over geological timescales (last ~200 million years) and their biogeochemical consequences.

In particular, the morphology of the intricate, individual calcite plates (coccoliths) that form a cell covering (the coccosphere) of coccolithophores have been shown to respond to a range of environmental perturbations in laboratory studies, including temperature [5–9], nutrient limitation [10–12], light intensity [11,12], carbonate chemistry [7,12–15], trace metals [12], and salinity [6,16–20]. Plankton populations and fossil assemblages also show variability in coccolith morphology and size spatially and on seasonal to geological timescales that have been linked to changing ocean conditions, e.g., [21–28]. Records of changes in coccolith morphology therefore make a valuable contribution to biogenic archives of past oceanographic conditions.

In contrast to research into the effects of temperature, nutrient availability and carbonate chemistry on phytoplankton ecophysiology, salinity has received rather limited attention in marine phytoplankton studies. Surface ocean salinity presently ranges between ca. 32 and ca. 38 in open ocean regions, with substantially more variability spatially and seasonally beyond these values in coastal, marginal and upwelling systems or more restricted basins [29,30]. There is evidence for global hydrological cycle intensification of 3–13% with every degree of global temperature increase [31] with associated observations of regional open-ocean salinity changes of up to ±0.2 over the past 50 years [32]. With changes in climate, local and regional ocean salinity may vary substantially on a range of timescales driven by altered precipitation, evaporation, terrestrial freshwater dynamics, sea ice melt, larger-scale atmospheric processes, and climate patterns such as monsoonal systems. Observations currently indicate that lower salinity (net precipitation) regions will likely freshen and higher salinity (net evaporation) regions will likely become increasingly saline under projected climate change scenarios [32,33]. Changes in sea surface salinity affect ocean circulation and stratification and will therefore contribute both directly and indirectly to the diverse effects of climate change on biological productivity through environmental impacts on physiology and shifts in ecological distributions e.g., [34]. In addition to the importance of understanding how salinity changes impact the ecophysiology of marine organisms, the ability to reconstruct past sea surface salinity conditions is also crucial for investigating variability in density-driven regional, global ocean circulation, and hydrological cycles through geological time [35] as well as the implications of these physical changes for biogeochemical cycling.

Previous studies on the response of coccolithophores to salinity have largely investigated the most abundant and widespread modern species *Emiliania huxleyi*, which exhibits a remarkable salinity tolerance across its global distribution. *Emiliania huxleyi* populations have been reported in salinities as low as 11–12 in coastal areas [18] and 18 the Black Sea [36], and as high as ca. 38–41 in the Red Sea [37] and other (semi-) enclosed basins ruled by high net evaporation (e.g., Mediterranean Sea, [38,39]). Such marginal/coastal systems and semi-enclosed basins commonly feature strong spatial salinity gradients and may experience more pronounced changes in mean and short-term salinity extremes over the coming century relative to open-ocean settings, as their evaporation-precipitation balance is particularly sensitive to the shorter-term hydrological cycle fluctuations that are likely to be amplified with climate change. Variations in *E*. *huxleyi* coccoliths observed along salinity gradients in sediment core top and plankton samples [16,40] and in culture [6,17–20] is of particular interest for investigating the productivity and carbonate production responses of past *Emiliania* populations to regional salinity changes (potentially over the last ~270 kyrs since its first occurrence [41,42]). Shifts in coccolith morphology (notably coccolith size) are also a useful contribution to the paleoceanographic toolkit for paleosalinity reconstructions. In particular, the paleosalinity proxy based on *E*. *huxleyi* coccolith size [16] is independent of other biological and geochemical proxies for paleosalinity, which makes it especially useful in marine sedimentary records where other paleosalinity proxies have relatively large uncertainties or cannot be applied (e.g., [43–46]). Recently, *E*. *huxleyi* coccolith size records have contributed paleosalinity proxy data to shed light on Holocene paleoceanographic conditions in the Mediterranean Sea and Black Sea region [47]. This illustrates their potential to complement geochemical reconstructions that highlight considerable regional salinity variability during intervals of higher- and lower-than-modern mean global paleosalinity in the geological record, for example during glacial periods [48,49] when salinity in the Red Sea may been as high as 50–55 due to changes in sea level and hydrological cycles [50–52].

Here, we assess whether the response of *E*. *huxleyi* to salinity change is variable across different strains isolated from a range of different salinity environments and coastal or semi-enclosed settings in addition to open-ocean strains. Specifically, we address the following questions: (i) is there a uniform response in growth rates to low and high salinity conditions across *E*. *huxleyi* strains isolated from a range of natural salinity environments? (ii) are changes in coccolith size and morphometry under low and high salinity conditions comparable between a strain isolated from a lower salinity environment (coastal Norwegian waters) *versus* a strain isolated from a higher salinity environment (Western Mediterranean Sea)? (iii) what associated changes in coccosphere size are observed in response to low and high salinity exposure in these two strains? and (iv) how strain-specific is the response of *E*. *huxleyi* coccolith length to low and high salinity and could this have implications for regional carbonate production and our interpretation of fossil assemblage records? Our study contributes to a growing body of evidence that there is potential for ecotype-specific responses to environmental perturbations, with consequences for both the past and future ecological and biogeochemical role of coccolithophores under different climate states.

## Methods

### *Emiliania huxleyi* strains

The growth response of *E*. *huxleyi* to three different salinity conditions was investigated across six clonal strains selected to represent a range of salinity environments (Table 1): PLYB11 (Plymouth Algal Culture Collection, Plymouth, UK), RCC1232, RCC1824, RCC1210, RCC868 and RCC904 (Roscoff Culture Collection, Roscoff, France). Furthermore, we investigated the morphological response to salinity in two of these six strains, which were selected to represent a contrast between a low salinity environment origin and high salinity environment origin (Table 1) that are representative of the end-members of the typical range in modern open-ocean sea-surface salinity (ca. 29 to 38): PLYB11 was isolated from coastal North Sea waters near Bergen, Norway (60° 18’N, 05° 15’E) with a mean annual salinity of 30–33 [29]; RCC1232 was isolated in the Mediterranean Sea (43° 41’N, 07° 19’E) with a mean annual salinity of ~37 [29]. As a lower salinity representative strain for morphological analysis, RCC1232 was preferred to RCC1210 isolated from the Baltic Sea, which is brackish and not considered to be representative of shelf seas in general (e.g., [53]) and salinities of ca. 20 are less representative of fully marine settings which may confine any further applicability of results to estuarine populations only. Stock cultures of all strains were maintained at a salinity of 35, a temperature of 15°C, and at a 12 hour light, 12 hour dark light cycle under an irradiance of ~70 μmol photons m^-2^ s^-1^ as determined by a Quantum Sensor (LI-COR Biosciences GmbH). Both stock and experimental cultures were grown in sterile artificial seawater medium that was prepared by diluting synthetic sea salt (Ultramarine, Waterlife Research Industries Ltd., UK) into distilled water with the addition of 0.5 g L^-1^ Tricine to prevent salt precipitation during autoclaving. At salinity 35, the artificial seawater medium prepared using this synthetic sea salt contained ca. 20 g L^-1^ chloride, ca. 1300 mg L^-1^ magnesium, and ca. 430 mg L^-1^ calcium (Ultramarine, Waterlife Research Industries Ltd., UK). After sterilization, the artificial seawater was enriched with trace metals and vitamins according to f/2 medium (Sigma-Aldrich; [54]).

**Table 1:** List of *Emiliania huxleyi* strains cultured in this study.

Strain name	Latitude	Longitude	Ocean/Sea	Salinity at isolation	Experiment
PLYB11	60° 18’ N	05° 15’ E	North Sea	~30	Growth + coccolith and coccosphere morphometry
RCC1232	43° 41’ N	07° 19’ E	Mediterranean Sea	~37	Growth + coccolith and coccosphere morphometry
RCC1824	33° 37 N	32° 39’ E	Mediterranean Sea	~39	Growth
RCC1210	59° 77’ N	20° 64’ E	Baltic Sea	~20	Growth
RCC868	31° 41’ S	91° 29’ W	South Pacific Ocean	~34	Growth
RCC904	39° 7’ N	14° 4’ E	Mediterranean Sea	~37	Growth

### Salinity experiments

We performed experiments at three salinities–a ‘low salinity’ treatment of 25, an ‘ambient salinity’ control of 35, and a ‘high salinity’ treatment of 45. These three salinity conditions encompass the global range of open ocean sea surface salinities, which range from a minimum of ~29 to a maximum of ~38 [29]. These salinity treatments also span the very low salinities present in coastal regions with large freshwater inputs as well as the very high salinities recorded in restricted, evaporation-dominated basins (for example salinities of ~41 observed in the northern Red Sea [55]). For strain RCC1210 from an isolation salinity of ~20 (Table 1), the ‘low salinity’ treatment of 25 is not a true low salinity condition in regard to its isolation from the much lower marine salinities present in the Baltic Sea, which is a marginal sea heavily influenced by freshwater inputs [56]. All six strains were maintained in stock culture under salinity 35 for several months prior to the start of the experiments.

Each salinity medium was prepared by diluting sterilized salt solution described above with sterile distilled water (prior to the addition of f/2 media enrichment solution) until the desired salinity was reached, as determined by a portable conductivity meter (WTW Multi 3400i, Xylem Analytics, Germany). Experiment cultures for each salinity treatment were grown in triplicate flasks under continuous (24 hour) light at an irradiance of ~70–100 μmol photons m^-2^ s^-1^ in 70 mL sterile polycarbonate flasks with ventilated lids to aid gas equilibration. Continuous light was used to desynchronize the timing of cell division to ensure that coccosphere size data were not influenced by cell cycle phase [57]. Each flask contained 60 mL of salinity 25, 35, or 45 media that was directly inoculated (no period of pre-experiment acclimation) with exponentially growing cells from salinity 35 stock cultures that had been maintained in semi-continuous batch culture prior to the start of the experiments. The absence of a pre-experimental acclimation phase allowed the observation of growth and morphological responses to an abrupt onset of low or high salinity conditions, which may occur naturally in the event of severe hydrological conditions or glacial/icesheet meltwater events for example (see Discussion). After inoculation each experimental flask had an initial concentration of approximately 2x10^5^ cells mL^-1^. Cultures were gently mixed daily to keep cells in suspension and flask headspace was able to re-equilibrate daily when flasks were opened under a laminar flow hood (sterile environment) to enable cell counts to be performed.

The pH and total alkalinity (TA) were measured at the start and end of the experiment for strains PLYB11 and RCC1232 (on which morphological analyses were perform). The pH was measured using a portable pH meter (WTW Multi 3400i, Xylem Analytics, Germany) and TA was measured using a titration method (MQuant Alkalinity Test, Merck). Dissolved inorganic carbon (DIC) was calculated approximately as the difference between the two acid capacity values determined through titration (KS4.3 –K_S8.2_). The measured and calculated carbonate system data are reported in S4 Table.

### Cell abundance and calculation of growth rates

**Fig 1:**
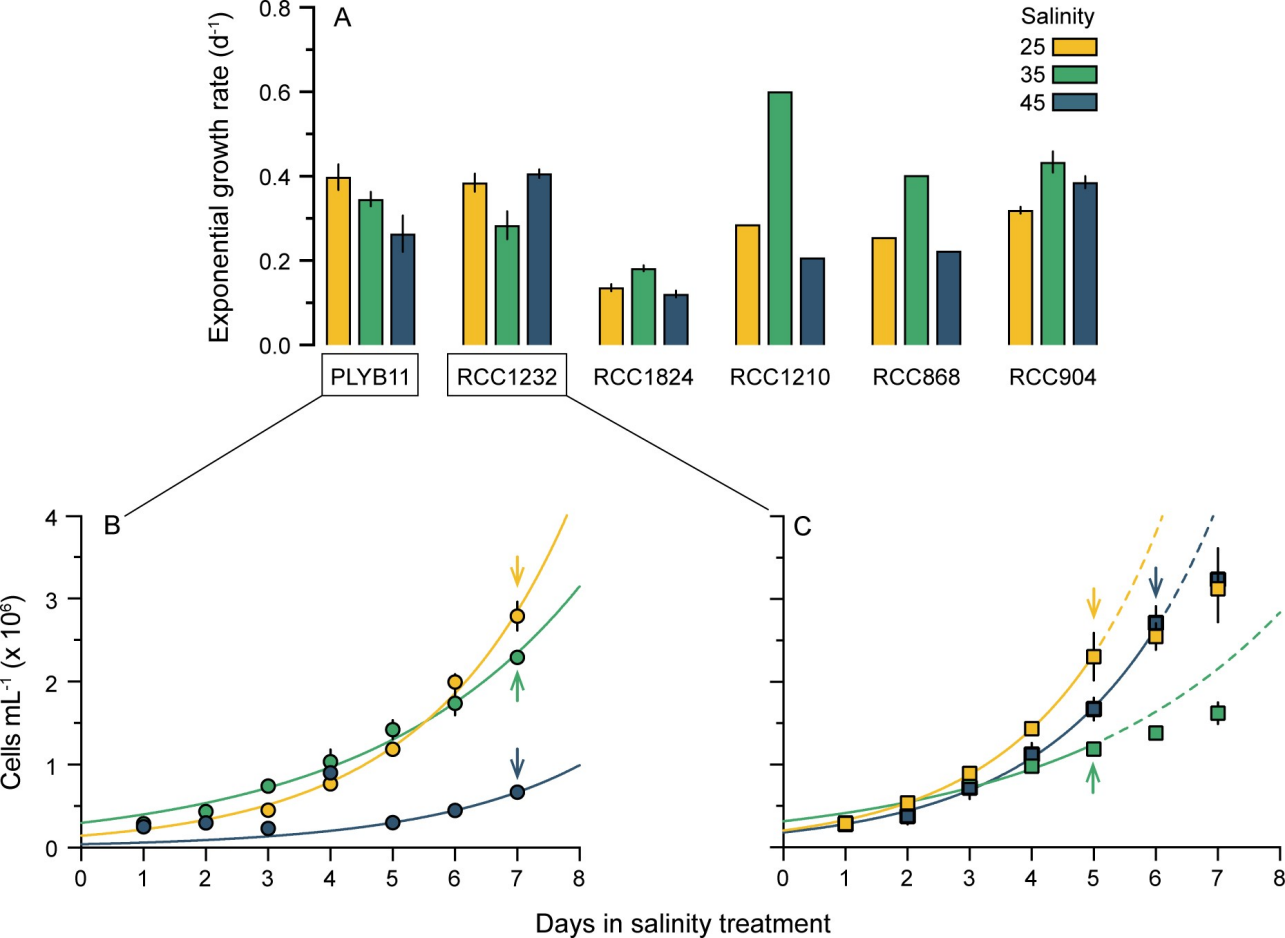
Growth rates of of six strains of *E*. *huxleyi* under three salinity conditions. (A) Exponential growth rates (mean and standard deviation, n = 3 for strains PLYB11, RCC1232, RCC1824 and RCC904; n = 1 for strains RCC1210 and RCC868) at salinity 25 (yellow), 35 (green) and 45 (blue). Detail of cell density with experiment day under each salinity condition for the two focal strains in this study: (B) strain PLYB11 and (C) strain RCC1232. Each data point represents the mean and standard deviation (bars) of cell counts from triplicate flasks. Lines (solid colour) show the exponential growth curve of each experiment fitted to exponential days of growth to indicate the point at which cultures stopped growing exponentially (days of non-exponential growth, dashed lines in C represent hypothetical continuation of exponential growth). The arrows indicate the day on which samples were taken for morphological analysis, with arrow colour corresponding to salinity treatment. See S1 Table for exponential growth rate data for each strain.

Cell density was quantified daily and used for the calculation of growth rates (Fig 1; S1 Table). Cell density was measured in triplicate for strains PLYB11, RCC1232, RCC1843 and RCC904 but quantified for only one of the three flasks for strains RCC1210 and RCC868. No statistical analyses were performed on the data for these two strains. To quantify cell density, flasks were inverted several times to ensure a homogenous suspension of cells and cell density was immediately measured from a 400 μL aliquot of experimental culture (dilution factor of 26) using a CASY Model TT cell counter (OMNI Life Science) fitted with a 60 μm capillary. The quantification size range for enumeration of cell density was 3.00 to 20.03 μm, which had been previously determined from all strains prior to the experiments. Exponential growth rates were calculated for each treatment flask as μ = ln(N_2_/N_1_)/(d_2_-d_1_), where N_2_ and N_1_ are the cell density on days d_2_ and d_1_, respectively. Mean exponential growth rates were then calculated as the average exponential growth rate across the triplicate flasks of each experiment for strains PLYB11, RCC1232, RCC1843 and RCC904. Exponential growth rates are based on single flask counts rather than triplicate flasks for RCC1210 and RCC868. Cell density and growth rate data for each strain are reported in S1 Table. The least-squared fit exponential growth curve for each salinity treatment for strains PLYB11 and RCC1232 (Fig 1B and 1C) was fitted to the mean (triplicate flasks) cell density during exponential days of growth under each treatment as follows: PLYB11, all salinity treatments = D1 to D7, RCC1232 salinity 25 and 35 treatments = D1 to D5, and salinity 45 treatment = D1 to D6.

### Sampling for morphological analyses

Whilst still in exponential growth, 2 to 5 mL of experiment culture from strains PLYB11 and RCC1232 were filtered onto polycarbonate filters (0.8 μm pore size, 25 mm diameter) using a borosilicate vacuum filtration flask (Millipore) and air dried for 24 hours. One filter was taken from each triplicate flask and morphological measurements (see section below) were performed on one of these triplicate samples from each salinity treatment. Samples for morphological analysis were taken when cultures had been growing under the salinity treatments for 5 days or longer but before cultures were expected to enter early stationary phase, therefore sampling occurred between day 5 and day 7 depending on the treatment and strain. For all PLYB11 salinity treatments, samples for morphometry were taken on day 7 and for RCC1232, sampling was done on day 5 for salinity 25 and 35 treatments and on day 6 for salinity 45. At the time of exponential sampling, cell densities ranged from 0.67x10^6^ to 2.8x10^6^ cells mL^-1^ (Table 2). Although sampling densities of ~1x10^5^ cells mL^-1^ or less are typically reported for *E*. *huxleyi* sampling (e.g., [58]), cultures were growing exponentially at the time of sampling (Fig 1) and there was no clear correlation between coccolith length and cell density or carbonate chemistry at the time of sampling. Each filter was mounted onto an aluminium stub and sputter-coated with 4-nm platinum in preparation for scanning election microscopy (SEM).

**Table 2:** Mean coccolith morphometry and coccosphere size, cell density at time of sampling, and growth rate in *Emiliania huxleyi* strains PLYB11 and RCC1232 grown at salinities of 25, 35, and 45.

	Salinity	Coccolith length (*C*_*L*_), μm			Coccolith width (*C*_*W*_), μm			Coccolith surface area (*C*_*SA*_), μm			Coccosphere diameter (Ø), μm			Cells density, mL^-1^ (x10^6^)		Growth rate, d^-1^	
		Mean	SD	n	Mean	SD	n	Mean	SD	n	Mean	SD	n	Mean	SD	Mean	SD
**PLYB11**																	
	**25**	2.52	0.29	56	1.92	0.27	56	3.85	1.06	56	4.22	0.45	53	2.8	0.20	0.40	0.03
	**35**	2.57	0.34	55	1.96	0.27	55	4.02	1.06	55	4.65	0.41	58	2.3	0.08	0.35	0.02
	**45**	3.19	0.46	62	2.57	0.41	62	6.57	1.98	62	5.07	0.41	58	0.67	0.04	0.26	0.04
**RCC1232**																	
	**25**	2.82	0.32	55	2.31	0.32	55	5.19	1.35	55	-	-	-	2.3	0.30	0.39	0.02
	**35**	2.94	0.29	54	2.43	0.27	54	5.68	1.17	54	5.36	0.50	50	1.2	0.06	0.28	0.03
	**45**	3.14	0.29	57	2.64	0.27	57	6.55	1.20	57	5.31	0.37	54	2.7	0.20	0.41	0.01

SD = standard deviation, n = number of individual coccoliths or coccospheres measured.

### Coccolith and coccosphere morphometry

*Emiliania huxleyi* coccoliths are small (typically ~2.5 to 5 μm) and formed from delicate calcite crystals with a thickness of only ~0.06 to 0.09 μm [20]. We therefore used high resolution SEM techniques (here a theoretical resolution of 0.003 μm) to obtain accurate and precise measurements of coccolith and coccosphere morphometry from strains PLYB11 and RCC1232. For size calibration, a filter with 2 μm polystyrene calibration beads was used (Duke Standards Microsphere 4000 Series, certified batch number 4202–008, certified mean diameter 1.998 μm ± 0.016 μm) and a vertical and horizontal measure of bead diameter was averaged for each individual bead to correspond to the same method of measurement detailed in [59] and [60].

Images of a minimum of 50 individual (detached, flat-lying, and non-overlapping) coccoliths (1024 x 768 pixels) were taken at random across each sample filter using a field-emission SEM (Zeiss SIGMA VP) at a magnification of 20,000x for the measurement of coccolith length (*C*_*L*_) and coccolith width (*C*_*W*_). Coccolith surface area (*C*_*SA*_) was calculated from *C*_*L*_ and *C*_*W*_ using the calculation for surface area of a 2-dimensional ellipse: *C*_*SA*_ = π(*C*_*L*_/2)(*C*_*W*_/2). Each coccolith was classified as ‘normal’ or ‘abnormal’ to quantify the degree of malformation present in each sample. Coccoliths were considered to be ‘abnormal’ when rim formation was notably asymmetrical, only the inner tube cycle was present, or more than one of the coccolith elements were incompletely formed (terminated before the rim) or fused together. Examples of these features are illustrated in S1 Fig. Malformation analyses are inherently subjective but are a recognized approach to obtain quantitative data (e.g., [61,62]).

**Fig 2:**
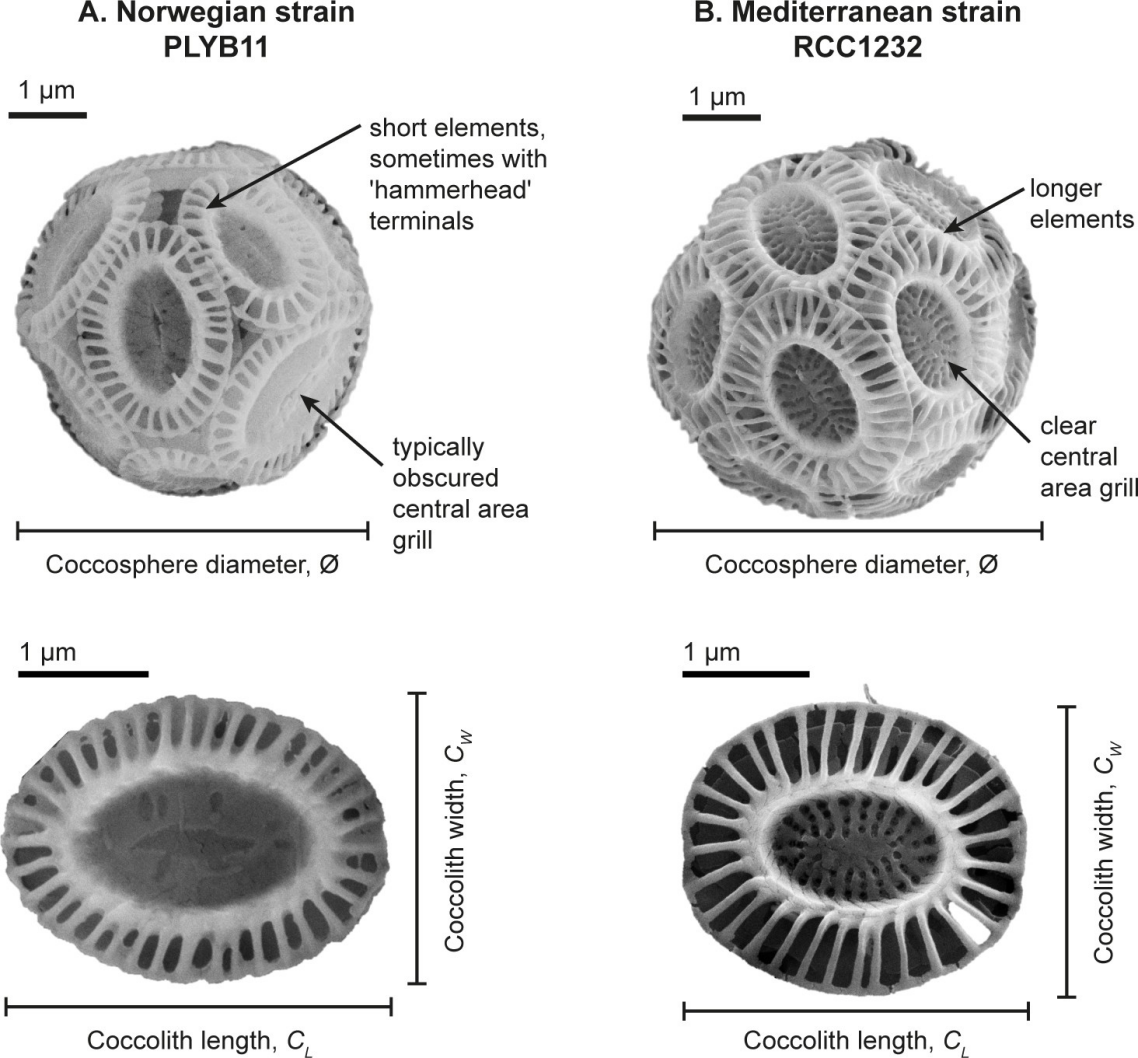
**Illustrative coccosphere (top) and coccolith (bottom) of *E*. *huxleyi*.** (A) strain PLYB11 and (B) strain RCC1232. Distinctive morphological features of each strain are highlighted and measurements of coccosphere diameter (Ø), coccolith length (*C*_*L*_) and coccolith width (*C*_*W*_) referred to in this study are defined. The scale bar represents 1 μm in all instances. All images are from salinity 35 treatment.

A minimum of 50 images (1024 x 768 pixels) of intact coccospheres (spherical cell covering formed from coccoliths) were taken at a magnification of 10,000x for measurement of coccosphere diameter (Ø), which here is the mean of one vertical and one horizontal measurement of size on each image for each of the >50 coccospheres measured on each sample (S2 Table). Fig 2 illustrates this suite of coccolith and coccosphere measurements. The morphometry of each coccolith and coccosphere was measured using the freeware ImageJ (v.1.51) calibrated to the calibration beads. Coccolith and coccosphere morphometry data for strains PLYB11 and RCC1232 are reported in S2 Table.

### Additional coccolith size data compiled from previously published literature

To further assess the degree of strain-specific morphological response to low and high salinity conditions, we present our new results within the context of data summarized from five previously published studies that collectively investigated morphological responses to salinity across 11 *E*. *huxleyi* strains—Paasche et al. (1996) [18], Green et al. (1998) [17], Fielding et al. (2009) [19], Saruwarti et al. (2016) [6], and Linge Johnsen et al. (2019) [20]. Each of these studies had performed experiments under very similar or identical salinity treatments to those used in our experiments–ca. 25, ca. 35 and ca. 45. Note that the highest salinity treatment in Fielding et al. [19] was 41 rather than 45. Other experimental conditions (temperature, light level and day length, seawater composition etc.) were variable from study to study and all published studies included a period of acclimation before the start of the experiments. Coccolith length data was the sole morphological variable to be consistently reported across all studies, so we retrieved data for mean coccolith length as reported at a specific salinity treatment in tables or results of the original manuscripts. Data that were not reported in a tabulated form or in the text were retrieved from publication data figures (e.g., scatter plots) using ImageJ software. The mean *C*_*L*_ data, number of originally measured coccoliths, and respective salinity treatment information are shown in S3 Table. From these data, we calculated the mean percentage decrease in coccolith length in response to low salinity 25 conditions [(salinity ca. 35 *C*_*L*_−salinity ca. 25 *C*_*L*_)/salinity ca. 35 *C*_*L*_]*100 and mean percentage increase in coccolith length under high salinity >40 conditions [(salinity ca. 45 *C*_*L*_−salinity ca. 35 *C*_*L*_)/salinity ca. 35 *C*_*L*_]*100 for each of the literature data (study and strain), relative to control salinity conditions of ~35. Coccolith length in these studies was originally measured from SEM images, with the exception of [20], where strain PLYB11 was measured by SEM and other strains were measured using circular polarized light microscopy. The samples analyzed by [20] were collected during an independent experiment with comparable experimental settings to the present study undertaken within our larger research programme on *E*. *huxleyi* responses to salinity, with the exception that the experimental flask volume was 20 mL rather than 70 mL.

### Statistical analysis and data visualization

Statistical analyses were performed using GraphPad Prism for macOS (v8.4.1, GraphPad Software, LLC) on growth rate data for strains PLYB11, RCC1232, RCC1843 and RCC904 and morphometry data for strains PLYB11 and RCC1232. The effect of salinity treatment on morphometry within PLYB11 and RCC1232 (e.g., *C*_*L*_ PLYB11 at 25 *versus C*_*L*_ PLYB11 at 35) and between strains at the same salinity treatment (e.g., *C*_*L*_ PLYB11 at 25 *versus C*_*L*_ RCC1232 at 25) was assessed using unpaired, two-tailed *t* tests that assumed a Gaussian distribution and were considered significant at the 95% confidence interval (p <0.05). Where percentages are reported, the mean values and original data are reported in the Tables and Supporting Information. Data figures were plotted in GraphPad Prism and final layout was arranged using Adobe Illustrator.

## Results

Under the control salinity conditions (salinity 35), mean exponential growth rates varied between the six strains (Fig 1A; S1 Table), with RCC1824 growing most slowly (μ = 0.18 d^-1^, SD = 0.008, n = 3) and RCC1210 growing most quickly (μ = 0.60 d^-1^, based on cell counts from a single flask). We note that the growth rates in RCC1824 at all salinities (0.12 to 0.18 d^-1^) are generally low for this species but not inconceivable [63] and are comparable to growth rates of 0.18 d^-1^ at salinity 25 and 0.19 d^-1^ at salinity 45 previously reported for this strain in a comparable experiment [20]. However, the growth rate at salinity 35 is ca. 40% lower than reported in [20], which may suggest that this strain was experiencing an unrecognized physiological stress prior to the start of the experiment that may have caused decreased growth rates. All strains grew under both the low salinity 25 and the high salinity 45 treatments, despite their direct (non-acclimated) exposure to the new conditions from salinity 35. In four of the six strains (RCC1824, RCC1210, RCC686, and RCC904), growth rate was highest at salinity 35 and decreased under both 25 and 45 salinity treatments. In contrast, growth rates in PLYB11 were significantly higher (μ = 0.40 d^-1^, SD = 0.03, n = 3) under the low salinity treatment (p = 0.0408, t = 2.979, df = 4) and significantly lower under the high salinity treatment (μ = 0.26 d^-1^, SD = 0.04, n = 3; p = 0.0372, t = 3.072, df = 4). In RCC1232, growth rates were significantly higher under both salinity 45 (p = 0.0036, t = 6.128, df = 4) and salinity 25 (p = 0.0111, t = 4.472, df = 4) compared to salinity 35 treatment (Fig 1).

**Fig 3:**
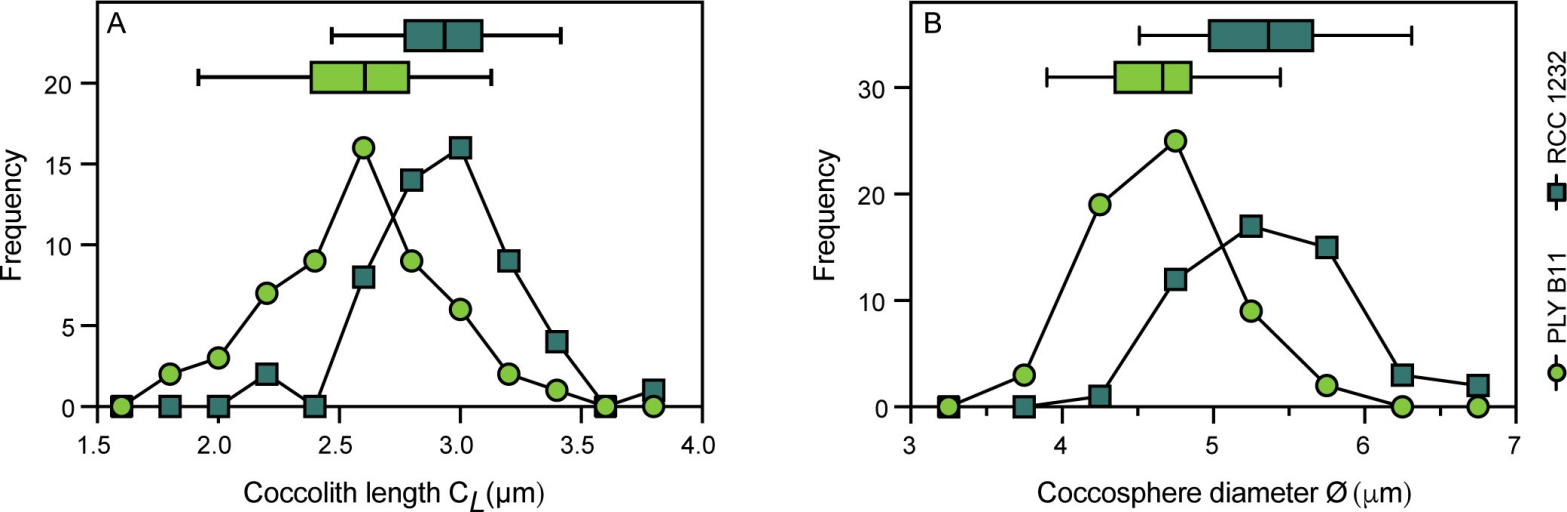
**Coccolith and coccosphere morphology of strain PLYB11 (green, circle) and strain RCC1232 (blue, square) grown at control salinity 35.** (A) Coccolith length (0.25 μm bin size) and (B) coccosphere diameter (0.5 μm bin size). Descriptive statistics (median, 10^th^, 25^th^, 75^th^, 90^th^ percentiles) of the data for each strain are presented above each histogram as box-and-whisker plots.

Strain differences in the response of coccolith and coccosphere morphology to low and high salinity conditions were investigated in strains PLYB11 and RCC1232 that were isolated from lower and higher salinity regimes, respectively (see Methods). Based on SEM imaging, both strains broadly exhibit ‘type A’ coccolith morphology (Fig 2; e.g. [64]). Mediterranean strain RCC1232 has robust distal shield elements that are roughly 50% of the distal shield area and a clearly defined central area grill. In contrast, the central area grill of coastal Norwegian strain PLYB11 is typically partially or completely obscured by an organic coating. PLYB11 has shorter distal shield elements relative to central area size and often exhibit ‘hammer head’ terminations, where the terminus of the shield elements has not fused with its neighbors to form a continuous circumference. Under control salinity 35 (Fig 3), morphometric data measured from SEM images shows that coccoliths of RCC1232 ranged in size from 2.18 to 3.79 μm (mean 2.94 μm) and were on average larger than those of PLYB11 (+14.6%, t = 6.106, df = 107, p<0.0001), which ranged between 1.73 and 3.40 μm in length. Coccoliths of RCC1232 were typically slightly less elliptical (calculated as *C*_*L*_/*C*_*W*_; S2 Table) with greater coccolith surface areas (overall +41%, t = 7.736, df = 107, p<0.0001) compared to PLYB11. Mean coccosphere sizes were also significantly larger at salinity 35 in RCC1232 (mean Ø = 5.36 μm) compared to PLYB11 (mean Ø = 4.65 μm; overall +15.3%, t = 8.124, df = 106, p<0.0001). Both *C*_*L*_ and Ø in these strains are approximately normally distributed (Fig 3).

**Fig 4:**
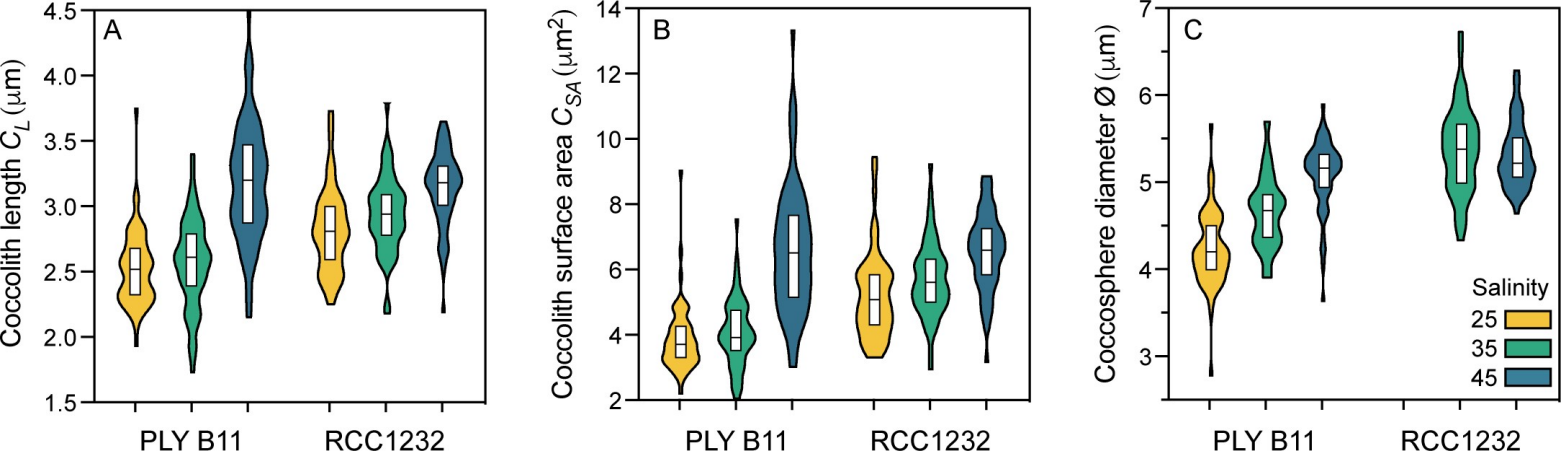
*Emiliania huxleyi* coccolith and coccosphere morphology under salinity conditions 25, 35 and 45. (A) Coccolith length, (B) coccolith surface area, (C) coccosphere diameter of *E*. *huxleyi* strains PLYB11 and RCC1232. Coccosphere diameter could not be measured in strain RCC1232 at salinity 25. Violin plots show the full range of morphological data with white 25^th^ to 75^th^ percentiles overlain. The central black line represents the median of the data. The mean, standard deviation and n of data are reported in Table 2 and full data in S2 Table.

In both strains, mean coccolith length (*C*_*L*_) was smaller under salinity 25 compared to 35 (statistically smaller in RCC1232, p = 0.0485, t = 1.996, df = 107; smaller but not significantly so in PLYB11). Both strains had significantly larger coccoliths under salinity 45 (PLYB11 p<0.0001, t = 8.271, df = 115; RCC1232 p = 0.0006, t = 3.547, df = 109) compared to cultures growing at 35 (Table 2; Fig 4). In PLYB11, the mean *C*_*L*_ increase from salinity 25 to 45 was 0.67 μm (26% increase). The mean *C*_*L*_ size increase observed between salinity 25 and 45 in RCC1232 was smaller, increasing by 0.32 μm (11.3%). Coccolith width (*C*_*W*_) had a positive relationship with *C*_*L*_ in both strains (not shown; S2 Table) and therefore increased or decreased proportionally with coccolith length under all treatments. This resulted in significant increases in coccolith surface area of 26.2% (p<0.0001, t = 5.647, df = 110) and 70.5% (p<0.0001, t = 9.160, df = 116) in strain RCC1232 and strain PLYB11, respectively, between the low and high salinity treatments (Fig 4B; S2 Table).

**Fig 5:**
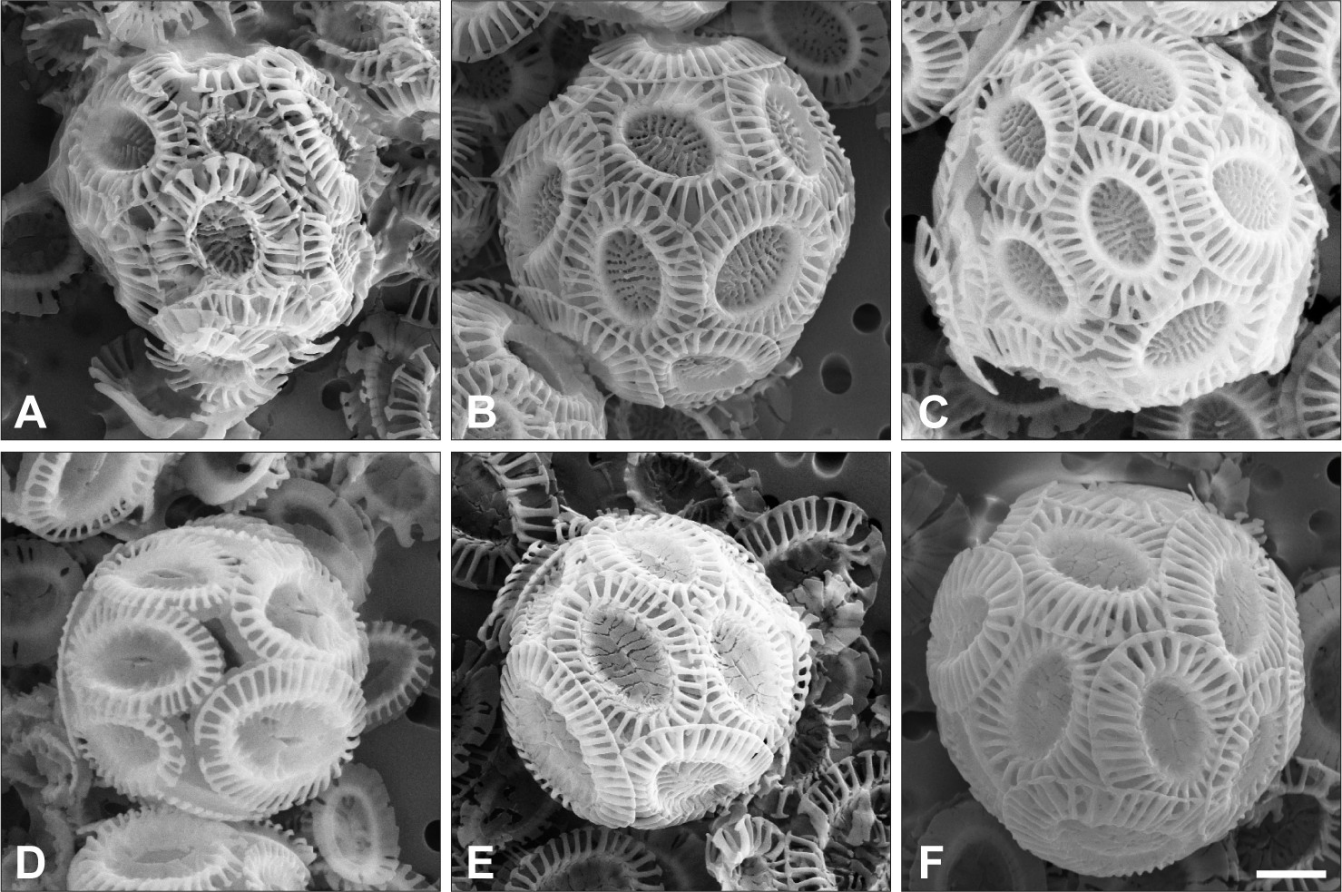
Representative *E*. *huxleyi* coccosphere morphology under each salinity treatment. Strain RCC1232 grown under (A) salinity 25, (B) salinity 35, and (C) salinity 45. Strain PLYB11 grown under (D) salinity 25, (E) salinity 35, and (F) salinity 45. All images are at the same scale, scale bar represents 1 μm.

In addition to significant within-strain changes in coccolith morphology, coccosphere size (Ø) also responded significantly to changing salinity (Table 2; Figs 4C and 5). In strain PLYB11, Ø was significantly smaller (mean 4.22 μm) at salinity 25 than at salinity 45 (mean 5.07 μm; p<0.0001, t = 10.54, df = 109). In strain RCC1232, there was no significant difference in Ø between salinity 35 and salinity 45 (t = 0.6742, df = 102, p = 0.5017). At salinity 25, no coccosphere measurements could be taken for RCC1232 because so few intact coccospheres were observed after filtration (Fig 5A).

## Discussion

**Fig 6:**
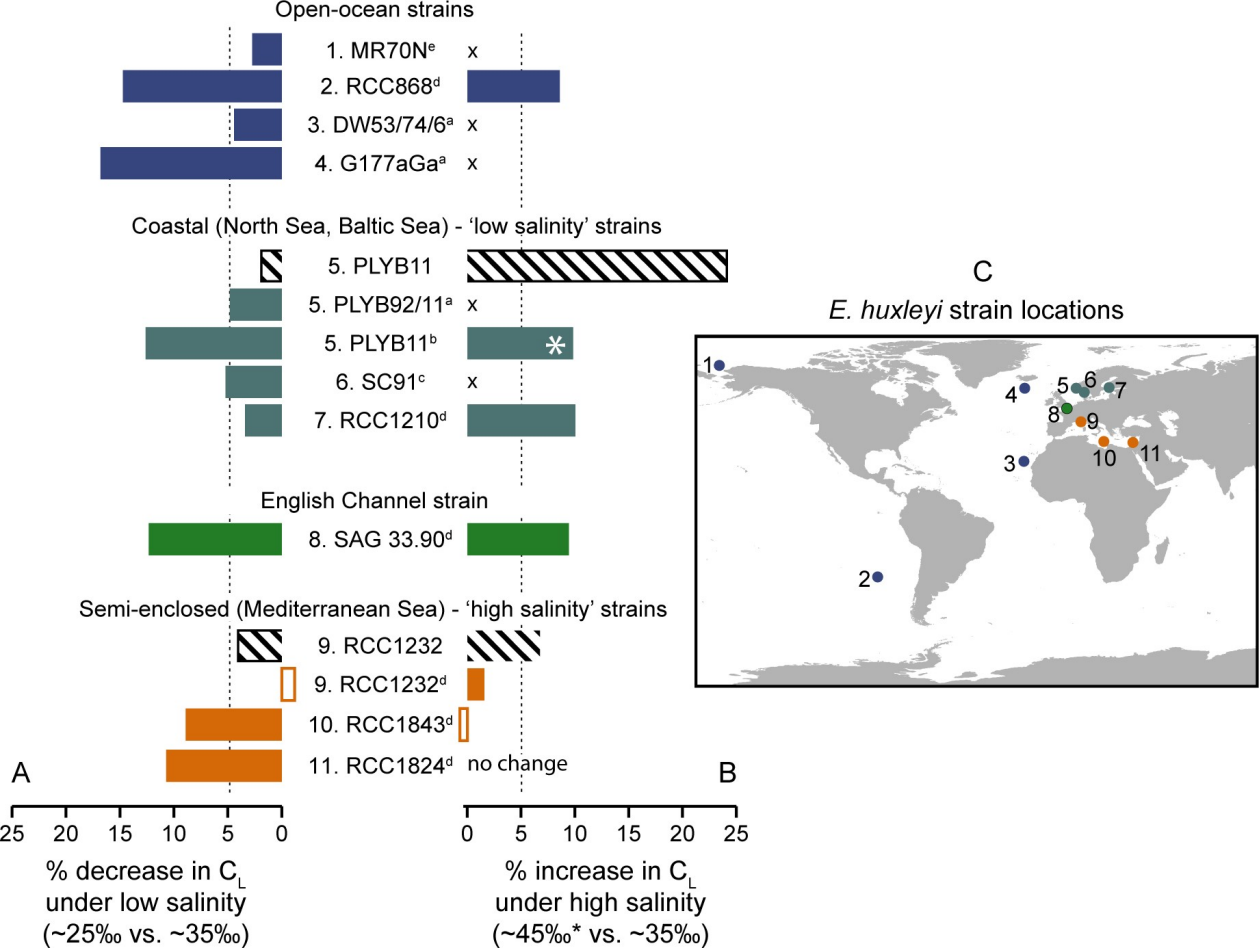
Comparison of coccolith size response (percentage change) across *E*. *huxleyi* strains under experimental high and low salinity conditions. (A) Percentage change in *C*_*L*_ under a salinity decrease from 35 to ~25, and (B) salinity increase from 35 to ~45 from strains PLYB11 and RCC1232 in this study (black stripe-fill bars) compared to results for a range of strains compiled from published literature (referenced by superscript letters: a. [17], b. [19], c. [18], d. [20], e. [6]). Strains have been numbered and are grouped respective to the salinity characteristics of their respective isolation locations, which are shown on map (C)–strain 1. MR70N, 2. RCC868, 3. DW53/74/6, 4. G177aGa, 5. PLYB11/PLYB92/11, 6. SC91, 7. RCC1210, 8. SAG33.90, 9. RCC1232, 10. RCC1843, 11. RCC1824. Where no high salinity experiments were undertaken for the strain, an ‘x’ is shown. Where the direction of *C*_*L*_ changes is opposite to the majority trend (i.e., a percentage increase in *C*_*L*_ where all other strains showed a percentage decrease in *C*_*L*_), the data bar is outlined without fill. The dashed vertical line in (A) and (B) denotes the 5% decrease or increase in *C*_*L*_, respectively, for guidance. The ‘*’ for the data PLYB11 from [19] indicates that the high salinity treatment in this study was at 41 rather than 45.

It has previously been shown that the growth and morphology of the coccolithophore *E*. *huxleyi* is sensitive to salinity conditions [6,16–20,40] that are likely to change with future climate change [31]. This may have biogeochemical consequences, as changes in morphology resulting from physiological responses to perturbed environmental conditions affects the calcite mass of individuals and therefore the production of calcium carbonate (e.g., [65,66]). The extent to which relationships between coccolithophore morphology and environmental stressors are species-specific is central to understanding the role of variable morphological traits and intraspecific diversity in the impacts of climate change (past and future) on biogeochemical cycles. To explore these themes, we investigate whether the response of *E*. *huxleyi* to salinity is strain-specific by comparing the growth and morphology of strains isolated from a range of salinity regimes and discussing our results within the context of available coccolith and coccosphere data from *E*. *huxleyi* strains from previously published research (Fig 6).

### The effect of rapid salinity change on morphology

An effect of salinity on coccolith length is observable even on short timescales, as we see differences in mean coccolith length of 11% and 24% in strains RCC1232 and PLYB11, respectively, after only 5–7 days growth under new salinity conditions. The direct exposure of experimental cultures to low and high salinity conditions from control conditions (without acclimation), the shorter-term duration of experiments in exponential phase of growth, and higher cell densities at sampling in our experiments records the most conservative degree of change in *E*. *huxleyi* coccolith morphology that might be captured by large, rapid-onset salinity changes. The higher cell concentrations at the point of sampling (Table 2; Methods) means that changes in the carbonate system (S4 Table) may have additionally contributed to the response of growth and/or morphology in these strains under these experimental conditions, particularly under salinity 25 where DIC concentrations changed by 18% (both strains) during the course of the experiment. It is also possible that the change in light conditions at the onset of the experiment (from 12:12 L:D to continuous light, see Methods) may have also contributed to the observed changes in morphology although, to our knowledge, there are no reports of changes in coccolith length due to changes in daylength.

The rapid onset of lower or higher salinity conditions may occur on meteorological timescales in near-shore, shelf sea, and marginal sea environments. For instance, sea surface salinity shifts of ca. 5 have been reported in the Bay of Bengal over timescales ranging from 4–5 days to a few weeks due to intense precipitation events associated with the abrupt onset of monsoon conditions [67] and the passage of tropical cyclones [68]. In open-ocean settings, short-term variability in sea surface salinity tends to be orders of magnitude smaller, for example a day-to-day salinity variability in the order of 0.03 to 0.07 in the Nordic Seas [30]. The effect of abrupt exposure to low and high salinity conditions over a period of 5–7 days on coccolith size are consistent with the response of coccolith size to comparable salinity treatments from previously published studies that had a period of pre-acclimation prior to the start of the experiment (Fig 6). This would support an interpretation that the response to a rapid change in salinity occurs quickly (within a few division cycles). *Emiliania huxleyi* would appear to be able to rapidly alter its physiological state in response to seawater salinity and this physiological response is directed at calcification processes as well as cell metabolism. Over longer timescales representative of hundreds of generations or years of exposure to new environmental conditions, it may be possible for *E*. *huxleyi* to show adaptation to permanent shifts in salinity conditions, as indicated by adaption of growth and calcification in this species to elevated CO_2_ conditions over hundreds of generations [69].

### Salinity effects on *E*. *huxleyi* morphology are strain-specific

Our measurements of *E*. *huxleyi* coccolith morphology under three salinity treatments (Fig 4) corroborates the trend of smaller coccoliths at lower salinities previously reported in culture experiments (Fig 6) [6,17–20]. Our results additionally show marked differences in the magnitude of response to low and high salinity conditions between the coastal Norwegian strain (PLYB11) and the Mediterranean strain (RCC1232). We also observed a significant trend in coccosphere size with salinity in PLYB11 (comparable data for RCC1232 could not be reported due to insufficient data at salinity 25; Fig 4C). The overarching trend of decreasing coccolith size with decreasing salinity that was first reported for plankton and sediment assemblages [16,40] is clear when available culture data for *C*_*L*_ under low and high salinity are synthesized from 11 *E*. *huxleyi* strains (Fig 6). However, the magnitude of response is not uniform across strains, with some strains showing only minimal responses (<5% size increase or decrease) and others changing by 10 to 24%. Additionally, three Mediterranean strains reported in [20] show negligible or very small (~1%) *C*_*L*_ changes opposite to this trend.

Overall, the strain-specific morphological responses reported here and across other strains from the literature (Fig 6) contribute to the well-documented variability in physiology reported across morphotypes and strains of *E*. *huxleyi* cultures from a wide range of environmental perturbations (e.g., [5,61,70,71]). The range of exponential growth rates across the six strains cultured here (Fig 1A) illustrates the physiological variability among strains growing concurrently under identical conditions. The mechanisms underpinning intraspecific differences in *E*. *huxleyi* physiology and its capacity to thrive under a wide range of habitats globally have been attributed to high diversity in its extensive genome [72]. The range of salinity environments (from ~20 to ~39) from which the strains here were isolated may have exerted genotype selection pressure on salinity tolerance. If this is the case, it is partly expressed in RCC1232 and PLYB11 as modified biomineralization processes and therefore quantifiable in changes in coccolith size. Whilst coccoliths of PLYB11 had a higher incidence of abnormality at salinity 25 (43% ‘normal’) compared to salinity 35 (69% ‘normal’; S1 and S2 Figs), any coccolith malformation did not result in noticeable consequences for coccosphere formation (Fig 5D) and growth rates were in fact highest at salinity 25 in this strain. The Norwegian coastal waters from which PLYB11 was isolated have a lower (~30–33) annual mean salinity that is likely to be further influenced by meteorological and seasonal variations in hydrological (fresher) runoff due to near-shore proximity [73]. In coastal fjords around the isolation location of PLYB11, *E*. *huxleyi* has been found to be growing, and even forming high concentration blooms, at low salinities of <25 ‰ [74,75] and multiple strains isolated from coastal waters in the North Sea and Baltic Sea were able to maintain better growth than open ocean strains at salinities as low as 12 ‰ [18,76]. Interestingly, Green et al. [17] showed that the same strain used here (in their study named PLYB92/11) continued coccolith formation at even lower salinities of 14 (with some, but not extreme malformation). Paasche et al. [18] also demonstrated that strain SC91, isolated from a similar locality to PLYB11 was similarly able to sustain calcification of abnormal but clearly recognizable coccoliths at extremely low salinities of 14. Our results are in agreement with these observations and suggest that PLYB11 is better adapted to lower salinity conditions, as growth rates are higher at salinity 25 and decline significantly at salinity 45 compared to growth at salinity 35. *C*_*L*_ changes are insignificant under low salinity exposure (35 to 25 conditions) but significant and large (24% increase) under high salinity conditions (35 to 45). Overall, it seems likely that strains from Scandinavian coastal environments, and therefore potentially others from similar coastal water settings, retain adaptations for low salinity tolerance and perhaps physiological plasticity under rapid changes in salinity, such as the conditions imposed in this study.

Malformations in natural extant coccolithophore populations have been regularly reported and linked to seasonal changes in salinity [77,78]. Generally, malformations are much more prevalent in culture experiments, where anywhere from <5% to ca. 30 to ca. 60% of coccoliths have been reported as ‘abnormal’ under control conditions. The percentage of coccoliths with malformations typically rises under environmental stress (e.g., [5,7,62,79]) and is more common in artificial seawater media [80]. Although coccoliths of strain RCC1232 showed comparable levels of malformation at salinity 25 (56% ‘abnormal’ coccoliths) to PLYB11 at salinity 25 (57% ‘abnormal’ coccoliths; S2 Fig) based on our classification criteria (see Methods), these malformations or the fragility of the coccoliths under low salinity conditions seems to have had a more significant impact in severely reducing the structural integrity of the coccospheres. In contrast, the incidence of malformation at salinity 45 was only slightly increased relative to salinity 35 conditions in this strain (24% ‘abnormality’ at salinity 35 *versus* 26% at salinity 45), whereas the incidence of ‘abnormal’ coccoliths rose from ca. 30% to ca. 50% in PLYB11 between salinity 35 and 45, respectively. Interestingly, in both strains the higher levels of ‘abnormal’ coccoliths at salinity 25 were associated with higher growth rates at salinity 25 than 35. This indicates that cellular calcification but not growth in strains RCC1232 isolated from higher salinity Mediterranean waters and PLYB11 from lower salinity Norwegian waters is sensitive to low salinity conditions on short timescales. RCC1232 was also the only strain of the six cultured to show an increase in growth rate at salinity 45 (compared to both salinity 35 and 25) indicating a greater tolerance of high salinity conditions than in the other strains, including Mediterranean strains RCC1824 and RCC904. The coccolith size response of RCC1232 to salinity extremes overall was smaller in magnitude than for PLYB11 despite the significant impact of low and high salinity on growth rates. A decrease in *C*_*L*_ of ~10% at salinity 25 (relative to 35) but negligible *C*_*L*_ change under high salinity was also reported in two other Mediterranean strains (RCC1824 and RCC1843 in [20]; Fig 6). There has been less research into the impact of increasing salinity on coccolithophores relative to low salinity effects. However, our results and data compiled from previous studies (Fig 6) provisionally indicate that some strains, but not necessarily all, from higher salinity regimes (like the Mediterranean Sea) may have a higher salinity tolerance and greater intolerance of low salinity conditions than their lower-salinity coastal counterparts.

Based on experimental exposure to low salinity conditions, it has also been suggested that open ocean strains may respond differently than strains from near-shore or marginal basin environments to changes in salinity [17,18]. Coccolith size decreases at low salinity were as high as ~15% in two open ocean strains [17,20] (Fig 6) and severe coccolith malformation at very low salinities of 16 was reported for two open-ocean strains (DW53/74/6 from the subtropical Atlantic and G177aGa from the North Atlantic [17]; see Fig 6C). It is feasible that the typical stability of physiochemical ocean conditions on shorter (sub-seasonal to annual) timescales in open-oceans would select for more physiological specialism given the more restrictive range of annual environmental conditions to avoid the energetic costs of plasticity that may lend a selective advantage under more dynamic nearshore environments [81]. This perspective would lend support to the earlier hypotheses of [40] that relationships between salinity and coccolith morphology in *E*. *huxleyi* are more likely than not related to the evolution of different physiological strategies for salinity stress in strains from different biogeographic regions (e.g., open ocean *vs*. coastal).

### Potential links between morphology and physiological responses to salinity stress

Coccolithophore cell, and by inference coccosphere, size is known to vary with the cell division cycle (e.g., [57]) and environmental conditions including nutrient availability, temperature, trace metals and CO_2_ (e.g., [7,82–85]). Cell volume in algae and other plants also responds to salinity changes by regulating cell turgor pressure to accommodate for changing ion gradients across cell membranes [86,87]. It has been suggested that (presumably active) cellular osmoregulation acts similarly in coccolithophores to alter cell volume and proportionally the size of the intracellular coccolith vesicle (where coccolith formation occurs), thus driving the relationship between salinity and coccolith size [40]. Here, some of the first data for the effect of salinity on the coccosphere (Figs 4D and 5) show an increase in coccosphere size with increasing salinity in strain PLYB11, but this was not seen clearly in RCC1232 (Fig 4D) as coccosphere size could only be compared between salinity 35 and 45 and no significant difference was observed.

The increase in coccosphere size (Ø) alongside increasing coccolith length as salinity increases in PLYB11 would initially appear to support the hypothesis put forward by Bollmann et al. [40] described above, that the effect of osmoregulation on cell volume may change the size of the coccolith vesicle and therefore *C*_*L*_. However, we consider that the mechanisms involved in coccolith size adjustment with salinity are not simply a function of Ø and are also likely to be strain-specific for the following reasons: we do not observe any clear change in Ø in RCC1232 between control (35) and high salinity (45) conditions, *C*_*L*_ decrease was conversely associated with Ø increase at low salinities in an open-ocean strain from the Chuckchi Sea (11% from 4.94 μm at 35 to 5.46 μm at 26) [6], and severe impairment of typical coccolith formation and calcification at low salinities is observed in some strains (RCC1232, this study and two open-ocean strains in Green et al., [17]) but not in others (PLYB11).

Salinity change clearly impacts coccolith size across most strains (Fig 6) and our observations indicated a greater incidence of coccolith malformations in both RCC1232 and PLYB11 in line with higher levels of malformations reported for other strains under low salinity conditions [17,18]. This indicates that salinity plays an important role in the cellular calcification process that is not yet well understood and seems to be expressed differently in different strains and somewhat decoupled from the effect of salinity on growth rates. Calcification (here referring to the intracellular formation of new coccoliths) has a very high energetic demand and is allocated ~20–40% of the total photosynthetic budget in *E*. *huxleyi* [88]. Smaller coccoliths and potential for coccolith fragility or malformation at low salinities could arise if the energetic costs of bicarbonate, carbonate and calcium ion uptake for calcification (‘transport’ costs; [88]) increased at lower salinities, where these ions tend to be available in lower concentrations. Conversely, larger coccoliths at high salinities may, in part, represent a strategy to expend excess carbonate ions. Changes to the energetic partitioning of calcification costs at lower salinities relative to other components of metabolic energy budgets have been reported for other marine calcifiers, including bivalves [89], although this is with the caveat that biochemical mechanisms of biomineralization are diverse across calcifiers (e.g., [90–92]).

We speculate that an unequal ability to regulate cell turgor through osmotic adjustment may contribute to the response of coccolith size to salinity and its strain-specific nature. In *E*. *huxleyi*, this process is likely to be related, at least in part, to strain-variable cellular accumulation of the osmoprotectant dimethylsulfoniopropionate (DMSP) [93–97] that is thought to be particularly important in algal physiology under long-term high salinity stress [86]. It has been demonstrated that levels of DMSP in *E*. *huxleyi* and other phytoplankton are variable and responsive to environmental perturbations that cause cellular oxidative stress [98]. Increases in cellular DMSP with salinity have been observed in the neritic coccolithophore *Hymenomonas carterae* [99] and in the marine diatom *Phaeodactylum tricornutum* [100]. If osmotic adjustment in *E*. *huxleyi* under salinity stress does lead to changes in cell volume, the magnitude of change and associated coccolith size response might be modulated by strain-specific DMS concentrations and, particularly, strain-specific capacity to increase DMS production under salinity stress. The plasticity of these protective biochemical pathways may be more likely to be selected for in some strains rather than others depending on the natural variability (magnitude and timeframes) experienced in their original isolation environment: a potential form of environmental ‘pre-conditioning’. Poorer osmoregulation ability in some strains may prompt trade-offs in energetic allocation to different cell processes that prove to be detrimental to coccolith mineralization. Future quantification of cellular DMSP quotas and other anti-stress osmolytes under a range of environmental conditions would be beneficial for understanding the biochemical processes underlying physiological responses to oxidative stress, which is expected to increase with climate change for most environmental stressors.

### Implications for biogeochemistry and interpretation of fossil assemblages

Carbonate production by coccolithophores is determined by growth rate and cellular (or population) calcite content. Cellular calcite is a function of coccolith volume (and therefore coccolith length) and the number of coccoliths per coccosphere [101]. The general trend towards larger *E*. *huxleyi* coccoliths under higher salinity regimes and smaller coccoliths under lower salinity regimes that we have observed (and has been reported for natural assemblages; [16,40]) may therefore cause a shift in the calcite content of populations. As an illustration of this, the 24% increase in *C*_*L*_ under salinity 45 relative to salinity 35 in PLYB11 observed here could hypothetically result in an approximate doubling in cell calcite content (19.7 pg cell^-1^ at salinity 45 *versus* 10.3 pg cell^-1^ at salinity 35) if all other parameters remained the same (calculated as cell calcite = *C*_*L*_^3^*K_S_*calcite density*C_*N*_ following [101], where K_S_ is a shape factor of 0.015 for *E*. *huxleyi* PLYB11, as determined experimentally by [20], and *C*_*N*_ is number of coccoliths per cell, arbitrarily set at 15 for this example). In light of the strain-specific response of growth rates and coccolith morphology to salinity change observed here, it is also feasible that changes in population carbonate production could also arise if the change in salinity conditions preferentially selected for ecotypes with better adaptations to the new salinity condition but with different coccolith morphologies (e.g., *C*_*L*_, thickness or morphotype). Any changes to the ratio between the production of calcite with respect to biomass (calcite: carbon) would have implications for the contribution of *E*. *huxleyi* to inorganic carbon export and whether *E*. *huxleyi* overall acts as a source or a sink of CO_2_ on a local scale [102]. These shifts might be most notable in marginal or restricted seas, where salinity conditions can be more dynamic on seasonal and decadal timescales and may respond more strongly to changes in regional and global hydrological cycles with climate change resulting in future salinity changes that may exceed the global average. For example, sea surface salinity in the Mediterranean Sea may be up to 0.9 higher than the 1961–1990 mean by the end of the 21^st^ century [103], due to decreased precipitation, increased evaporation, and the effect of human activity on freshwater inputs [104]. Cellular calcite mass also depends on coccolith thickness and morphotype characteristics in addition to coccolith length, and there is evidence that coccolith length and thickness may not increase proportionally in all *E*. *huxleyi* strains or show the same response to salinity [20]. However, changes in ocean salinity conditions must be considered alongside the effects of CO_2_, temperature and nutrients on the calcification and biogeochemical role of *E*. *huxleyi* under future climate conditions.

Shifts in the size of *E*. *huxleyi* coccoliths in fossil assemblages has been established through a number of studies as a useful independent proxy for sea surface salinity [16,19,40], most recently demonstrating sea-level-associated changes in outflow in the Black Sea region of the Mediterranean [47]. The results of our study are in agreement with global compilations of *E*. *huxleyi* mean coccolith size that show a clear relationship with in-situ salinity [16,40] but importantly show that this size response is strain specific and therefore likely to be linked to phenotypic or genotypic variability in *E*. *huxleyi*. The component of changes in fossil coccolith size within marine sedimentary sequences that is attributed to a salinity signal will therefore combine both size change from physiological adjustment (represented by within-strain results) and changing abundances and/or distributions of genotypically-distinct ecotypes with differences in morphology and morphological responses to environment (represented by strain-specific responses). A transfer function to determine paleosalinity from assemblage *C*_*L*_ has been developed for *E*. *huxleyi* from core top and plankton samples [16,40] and is supported by culture data [19,20]. However, the strain-specific response of *C*_*L*_ to salinity may well be related to the influence of the isolation environment on physiological plasticity to salinity change. As such, a globally-derived paleosalinity proxy based on open-ocean samples may not be universally applicable to fossil assemblages from all locations without further calibration based on the magnitude of *C*_*L*_ response typical of regional strains. However, the rapid response of *E*. *huxleyi* physiology and coccolith morphology to sudden changes in salinity conditions reported here is further evidence that *E*. *huxleyi* coccoliths in the fossil record will have been sensitive recorders of environmental changes on a range of timescales that have been integrated into the time-averaged *C*_*L*_ signal that can be measured from fossil assemblages (e.g., [47]).

## Conclusions

It is known that the coccolith morphology of the key calcifying phytoplankton species *E*. *huxleyi* responds to increasing salinity by increasing coccolith size in natural populations and in laboratory settings. Using culture experiments with two strains from contrasting salinity regimes synthesized with published size data from strains isolated from a range of salinity settings, we find that the magnitude of this salinity-driven *C*_*L*_ size change is strain-specific. We also find that coccosphere size increases with increasing salinity in strain PLYB11 from coastal Norwegian waters but not in Mediterranean strain RCC1232. The coccolith formation of RCC1232 was additionally severely affected under the low salinity treatment to the extent that coccosphere structural integrity was compromised, suggesting that the calcification physiology of this strain cannot adjust to such low salinity conditions. We hypothesize that these strain-specific responses arise from different adaptations to the natural salinity regime from which each strain was originally isolated. Given the abrupt exposure to low and high salinity conditions imposed in our experiments, we also speculate that strain differences in the ability to readjust the energetic costs of calcification and osmoregulation capacity, perhaps through strain variability in DMSP, may play a role in underpinning the sensitivity of growth and coccolith formation in different strains. Clearly, there is considerable scope for future investigations into the role of salinity in resolving the molecular mechanisms that govern biomineralization in coccolithophores and the biochemical variability in osmoregulation ability that may contribute to strain sensitivity or plasticity under shifts in salinity conditions. Strain-specific responses in growth and calcification have implications for regional carbonate production under future ocean conditions that may drive both within-ecotype size changes and shifts in the biogeography of ecotypes with different coccolith and coccosphere size distributions. Sea surface salinity conditions are spatially variable and future intensification of hydrological cycles is predicted to increasingly amplify these salinity features by freshening lower salinity regions, increasingly saline higher salinity regions, and contributing density-driven changes in stratification. Therefore, determining the response of marine phytoplankton physiology to salinity in addition to other, more commonly, investigated environmental variables like CO_2_ and temperature is a necessary contribution towards better understanding the future ecophysiology of marine primary producers.

## Supporting information

S1 Fig Examples of ‘normal’ and ‘abnormal’ coccoliths for *Emiliania huxleyi* strains RCC1232 (A. -D.) and PLYB11 (E.-H.), as defined in this study. A. ‘Normal’ coccolith (RCC1232 salinity 25); B. ‘Abnormal’ coccolith (RCC1232 salinity 25) showing incomplete coccolith elements; C. ‘Abnormal’ coccolith (RCC1232 salinity 35) showing incomplete coccolith elements; D. ‘Abnormal’ coccolith (RCC1232 salinity 45) showing incomplete coccolith elements and an asymmetric rim in the lower right side; E. ‘Normal’ coccolith (PLYB11 salinity 35); F. ‘Abnormal’ coccolith (PLYB11 salinity 25) showing incomplete and fused coccolith elements and distinct ‘hammer head’ terminations of elements that were common in this strain in all treatments; G. ‘Abnormal’ coccolith (PLYB11 salinity 35) showing incomplete coccolith elements; H. ‘Abnormal’ coccolith (PLYB11 salinity 45) showing fused and incomplete coccolith elements in the upper right side. Scale bar represents 1 μm and applies to all images.
(TIF)

S2 Fig Percentage of coccoliths classified as ‘normal’ and ‘abnormal’ for *Emiliania huxleyi* strains PLYB11 and RCC1232 under salinity 25, 35, and 45 conditions. Solid fill = ‘normal’ coccoliths, lined fill = ‘abnormal’ coccoliths.
(TIF)

S1 Table Mean exponential growth rates of six strains of *Emiliania huxleyi* at salinity 25, 35, and 45. (XLSX)

S2 Table Morphometric measurements of *Emiliania huxleyi* strains PLYB11 and RCC1232 under salinity 25, 35, and 45 conditions. (XLSX)

S3 Table Data on *Emiliania huxleyi* coccolith length under low and high salinity conditions compiled from previously published literature. (XLSX)

S4 Table Summary of carbonate system parameters at the beginning and end of experiment for *Emiliania huxleyi* strains PLYB11 and RCC1232. (XLSX)
