# Supplementary figures and images for: Strain-specific morphological response of the dominant calcifying phytoplankton species Emiliania huxleyi to salinity change
Source: PLoS One. 2021 Feb 11;16(2):e0246745. doi: 10.1371/journal.pone.0246745 (PMC7877742; doi:10.1371/journal.pone.0246745)

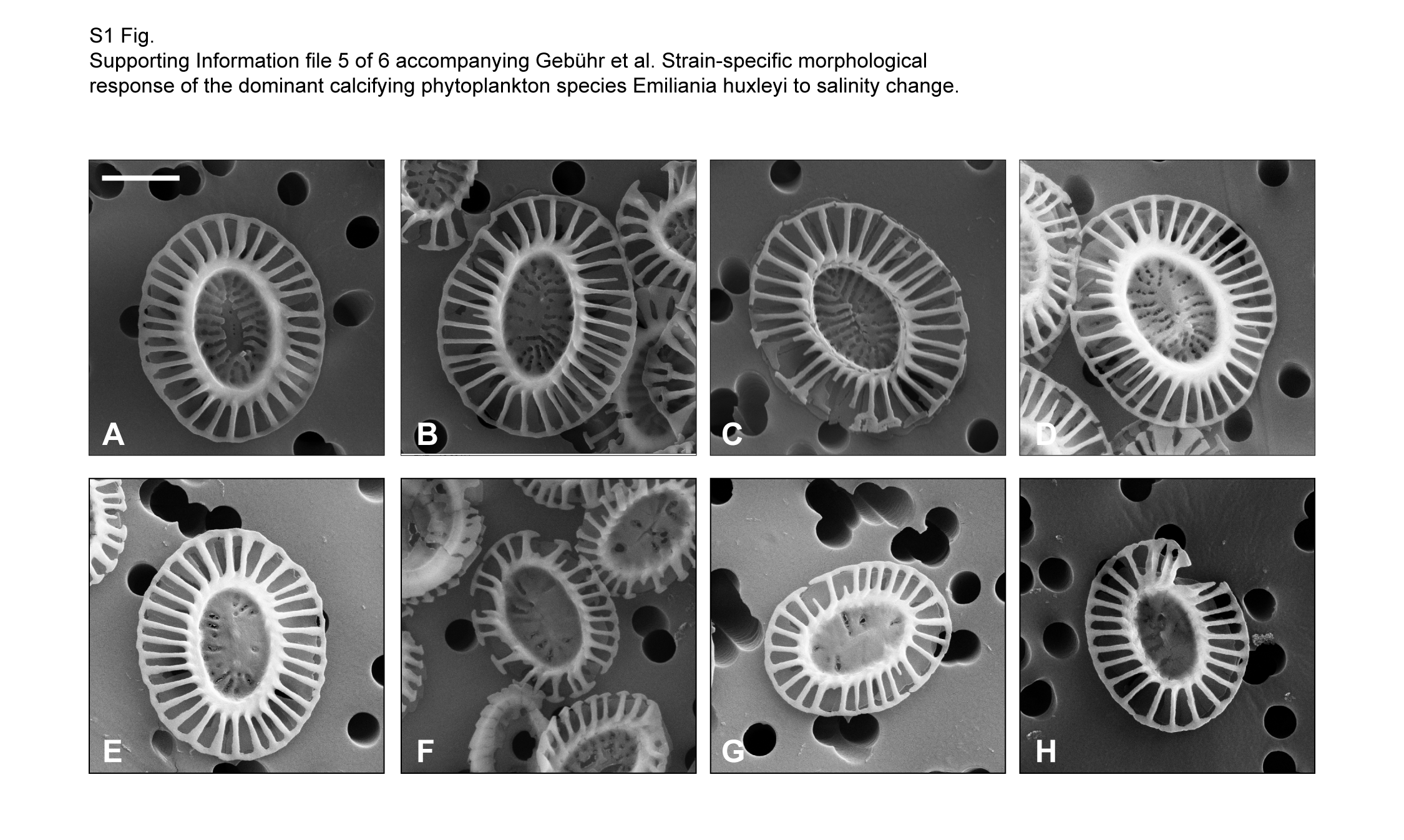

Supplement: S1 Fig — Examples of ‘normal’ and ‘abnormal’ coccoliths for Emiliania huxleyi strains RCC1232 (A. -D.) and PLYB11 (E.-H.), as defined in this study. A. ‘Normal’ coccolith (RCC1232 salinity 25); B. ‘Abnormal’ coccolith (RCC1232 salinity 25) showing incomplete coccolith elements; C. ‘Abnormal’ coccolith (RCC1232 salinity 35) showing incomplete coccolith elements; D. ‘Abnormal’ coccolith (RCC1232 salinity 45) showing incomplete coccolith elements and an asymmetric rim in the lower right side; E. ‘Normal’ coccolith (PLYB11 salinity 35); F. ‘Abnormal’ coccolith (PLYB11 salinity 25) showing incomplete and fused coccolith elements and distinct ‘hammer head’ terminations of elements that were common in this strain in all treatments; G. ‘Abnormal’ coccolith (PLYB11 salinity 35) showing incomplete coccolith elements; H. ‘Abnormal’ coccolith (PLYB11 salinity 45) showing fused and incomplete coccolith elements in the upper right side. Scale bar represents 1 μm and applies to all images. (TIF) [file pone.0246745.s001.tif]

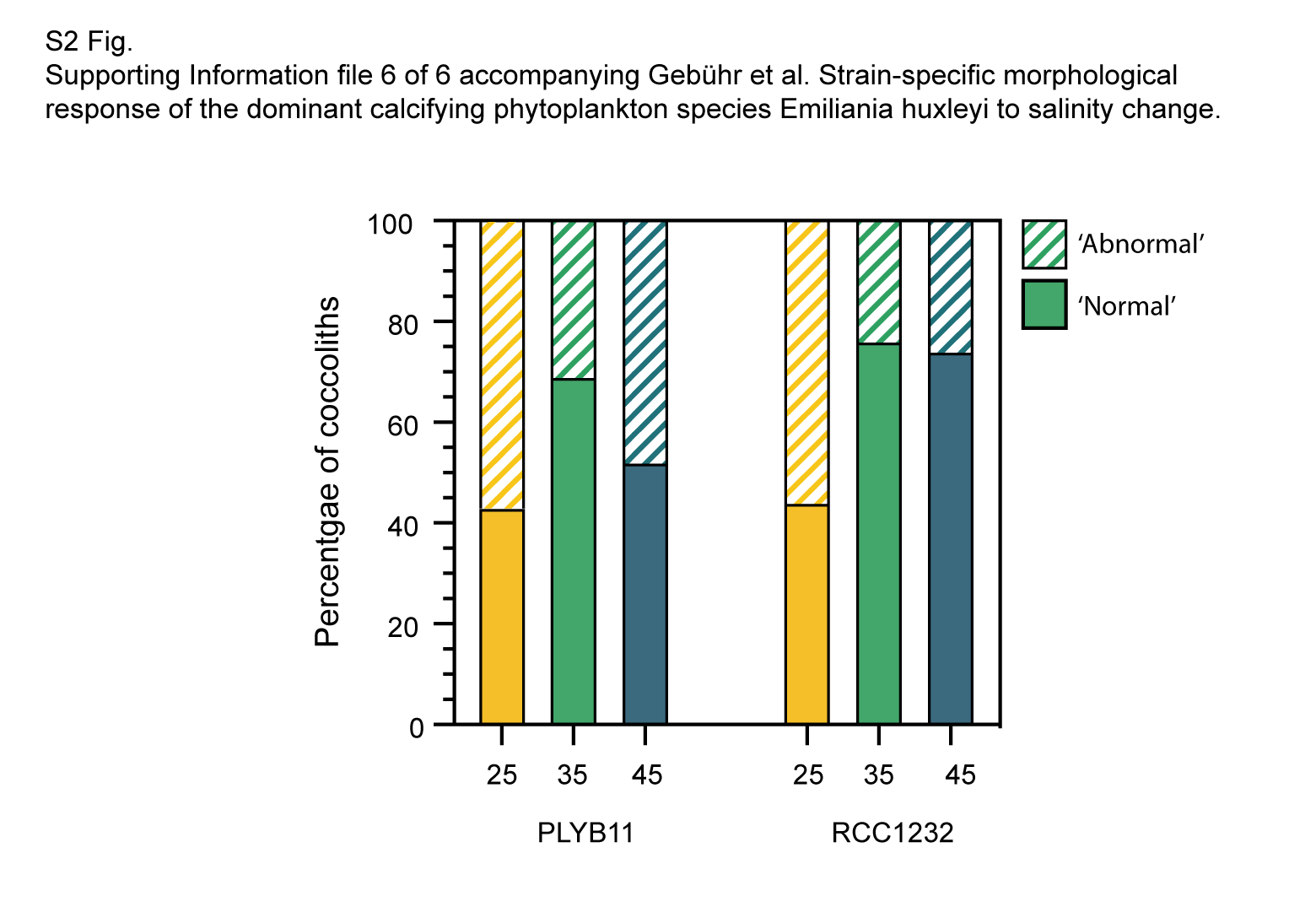

Supplement: S2 Fig — Solid fill = ‘normal’ coccoliths, lined fill = ‘abnormal’ coccoliths. (TIF) [file pone.0246745.s002.tif]
